# Both Boceprevir and GC376 efficaciously inhibit SARS-CoV-2 by targeting its main protease

**DOI:** 10.1038/s41467-020-18233-x

**Published:** 2020-09-04

**Authors:** Lifeng Fu, Fei Ye, Yong Feng, Feng Yu, Qisheng Wang, Yan Wu, Cheng Zhao, Huan Sun, Baoying Huang, Peihua Niu, Hao Song, Yi Shi, Xuebing Li, Wenjie Tan, Jianxun Qi, George Fu Gao

**Affiliations:** 1 CAS Key Laboratory of Pathogenic Microbiology and Immunology, Institute of Microbiology, Chinese Academy of Sciences, 100101 Beijing, China; 2 Center for Influenza Research and Early Warning (CASCIRE), CAS-TWAS Center of Excellence for Emerging Infectious Disease (CEEID), Chinese Academy of Sciences, 100101 Beijing, China; 3 NHC Key Laboratory of Biosafety, National Institute for Viral Disease Control & Prevention, Chinese Center for Disease Control and Prevention, China CDC, 102206 Beijing, China; 4 University of Chinese Academy of Sciences, 100049 Beijing, China; 5 Shanghai Synchrotron Radiation Facility, Shanghai Advanced Research Institute, Chinese Academy of Sciences, 201204 Shanghai, China; 6 Research Network of Immunity and Health (RNIH), Beijing Institutes of Life Science, Chinese Academy of Sciences, 100101 Beijing, China; 7 Department of Pathogen Microbiology, School of Basic Medical Sciences, Capital Medical University, 100069 Beijing, China; 8 Savaid Medical School, University of Chinese Academy of Sciences, 100049 Beijing, China

**Keywords:** Proteases, High-throughput screening, SARS-CoV-2, Nanocrystallography

## Abstract

COVID-19 was declared a pandemic on March 11 by WHO, due to its great threat to global public health. The coronavirus main protease (M^pro^, also called 3CLpro) is essential for processing and maturation of the viral polyprotein, therefore recognized as an attractive drug target. Here we show that a clinically approved anti-HCV drug, Boceprevir, and a pre-clinical inhibitor against feline infectious peritonitis (corona) virus (FIPV), GC376, both efficaciously inhibit SARS-CoV-2 in Vero cells by targeting M^pro^. Moreover, combined application of GC376 with Remdesivir, a nucleotide analogue that inhibits viral RNA dependent RNA polymerase (RdRp), results in sterilizing additive effect. Further structural analysis reveals binding of both inhibitors to the catalytically active side of SARS-CoV-2 protease M^pro^ as main mechanism of inhibition. Our findings may provide critical information for the optimization and design of more potent inhibitors against the emerging SARS-CoV-2 virus.

## Abstract (web summary)

Coronavirus main protease is essential for viral polyprotein processing and maturation. Here Fu et al. report efficient inhibition of SARS-CoV-2 replication using two inhibitors - Boceprevir and GC376 - targeting the active site of the main viral protease.

## Introduction

In December 2019, a novel coronavirus was discovered due to emerging viral pneumonia cases^1–5^. The virus has rapidly spread to more than 200 countries in the world^6,7^. The World Health Organization (WHO) named the infectious disease as COVID-19, and declared a global pandemic on 11 March 2020^8^. As of 28 March, the disease has caused more than 500,000 human infections with over 20,000 deaths globally^9^.

Human coronavirus 2019 (HCoV-19)^10^ is the seventh coronavirus capable of infecting humans, which was later named SARS-CoV-2 by the International Committee on Taxonomy of Viruses (ICTV)^11^ though with different name being proposed. The other six coronaviruses are the low-pathogenicity members including HCoV-OC43, HCoV-HKU1, HCoV-NL63, and HCoV-229E, and highly pathogenic SARS-CoV and MERS-CoV^12^. The clinical manifestations of COVID-19 include fever, fatigue, dry cough, headache, and diarrhea^13^. The median incubation period of the disease is 4 days, and the longest is no more than 41 days^13^. The patient at incubation period is contagious, and the median duration of viral shedding was 20 days in survivors, but the SARS-CoV-2 was detectable until death in non-survivors^14^. The longest observed duration of viral shedding in survivors was 37 days^14^. Some mildly ill patients do not have fever and obvious respiratory symptoms^14^. Most of the patients have a good prognosis, and the symptoms of children are relatively mild^14^. The patients with older age and comorbidities such as hypertension, diabetes, and coronary heart disease will have high risk of death^14^.

Sequence comparison has showed that the SARS-CoV-2 has the closest relationship (96.2%) with the bat SARS-like coronavirus^4,12^, but the origin of this virus was yet to be identified. Recent studies have shown that the SARS-CoV-2 uses the same Angiotensin-converting enzyme 2 (ACE2) receptor as SARS-CoV^4^, and the structural basis of receptor recognition was quickly elucidated to provide important basis for the molecular understanding of virus entry process and the development of potential antiviral inhibitors^15–18^. Some old drugs such as Remdesivir, Favipiravir, and Chloroquine/Hydroxychloroquine, and also traditional Chinese medicines showed potential for the treatment of COVID-19^19–22^. However, to date, no clinically approved specific drugs or vaccines are available to treat the disease. Therefore, it is urgent to develop specific drugs against the virus.

The coronavirus main protease (M^pro^, 3CLpro) is essential for viral polyproteins processing and maturation, therefore, it is recognized as an attractive drug target. Actually, viral proteases are also promising targets for many different viruses including hepatitis C virus (HCV)and human immunodeficiency virus (HIV)^23^. As there is neither specific antiviral agents nor available vaccines, repurposing of clinically approved drugs to combat the COVID-19 is urgently needed. Here we show both Boceprevir and GC376 can inhibit M^pro^ activity and SARS-CoV-2 in Vero cells. Moreover, combination of GC376 with Remdesivir treatment can enhance antiviral activity. Additionally, the high-resolution crystal structures of SARS-CoV-2 M^pro^ complex with these two inhibitors are solved to figure out their mechanism of inhibition. Taken together, these data provide critical information for the optimization and design of more potent inhibitors against the emerging pathogen SARS-CoV-2.

## Results

### High throughput drug screening

**Fig. 1:**
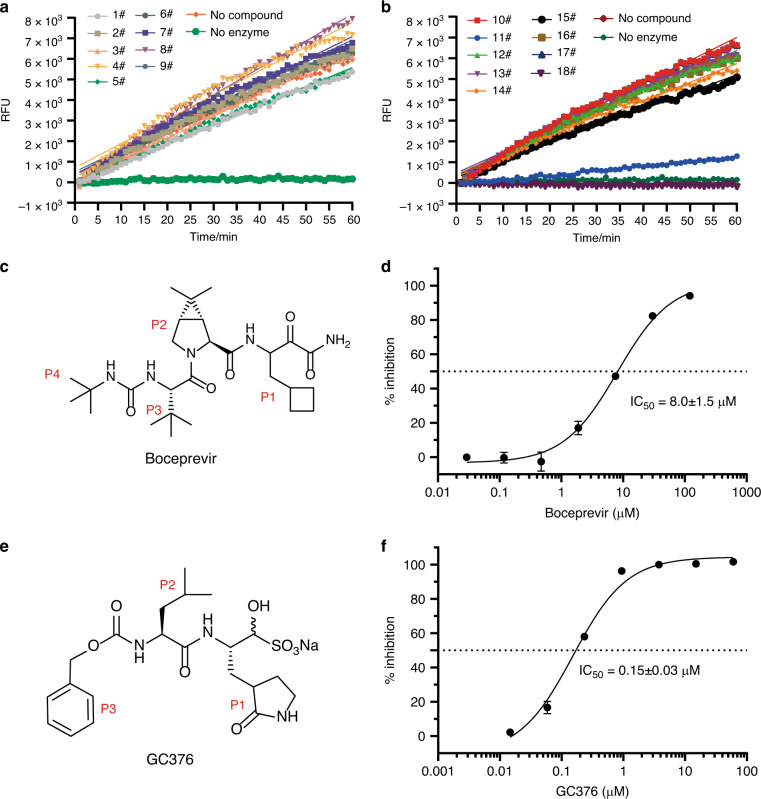
Boceprevir and GC376 can inhibit the catalytic activity of SARS-CoV-2 M^pro^ in vitro. **a**, **b** The initial screening assay for these compounds. Chemical structure of Boceprevir (**c**) and GC376 (**e**). **d**, **f** The inhibitory assay of Boceprevir (**d**) and GC376 (**f**) show efficient inhibition for M^pro^. Error bars: mean ± S.D. of three independent replicates. Source data are provided as a source data file.

In the beginning, we obtained soluble and pure M^pro^ protein of the SARS-CoV-2 with an extra glycine residue at the N-terminus (GM^pro^) after TEV cleavage by expression in *E. coli* cells. At the same time, we found that the M^pro^ of SARS-CoV-2 has high homology with other CoV M^pro^ (Supplementary Fig. 1). So, we selected a cluster of 18 chemical drugs that were designed to target the different viral proteases and proteasome (Table 1). Then, we screened these chemical drugs by in vitro fluorescence resonance energy transfer (FRET) enzymatic assays at a single concentration (100 μM; Fig. 1a, b). We identified two inhibitors, Boceprevir and GC376, can inhibit the enzymatic activity well (Fig. 1b). In contrast, other drugs such as Aluvia^®^ (HIV protease inhibitors, lopinavir, and ritonavir) did not show detectable inhibitory activity, which is consistent with the reports of recent clinical trials that Aluvia^®^ has no obvious effect on the treatment of COVID-19^24^.

**Table 1:** Eighteen anti-proteinase compounds were selected for screening.

Number	Target	Drug name
1	HIV Protease	Saquinavir
2	HIV Protease	Ritonavir
3	HIV Protease	Indinavir
4	HIV Protease	Nelfinavir Mesylate
5	HIV Protease	Amprenavir
6	HIV Protease	Lopinavir
7	HIV Protease	Atazanavir sulfate
8	HIV Protease	Fosamprenavir
9	HIV Protease	Tipranavir
10	HIV Protease	Darunavir
11	HCV NS3 protease	Boceprevir
12	HCV NS3 protease	Telaprevir
13	HCV NS3 protease	Simeprevir
14	HCV NS3 protease	Asunaprevir
15	HCV NS3 protease	Grazoprevir
16	Proteasome	Carfilzomib
17	Proteasome	Bortezomib
18	3C-Like Protease	GC376 sodium

### Enzyme activity study and its structural basis

Before further evaluating protein level inhibitory activity of Boceprevir and GC376, we found that extra residues at the N-terminus would impair the enzyme activity in previous report^25^. Therefore, we expressed the native M^pro^ without any extra amino acids at the N-terminus (a gift from Dr Haitao Yang in ShaghaiTech University) according to the literature^26^. Then, we compared GM^pro^ activity with the native M^pro^. Substrate hydrolysis rate of the native M^pro^ (frist 1000 s initial speed *v*_0_ = 1618 ± 84 RFU s^−1^) is almost three times faster than that of GM^pro^ (first 1000 s initial speed *v*_0_ = 449 ± 19 RFU s^−1^), which must be caused by extra Gly at the N terminus (Supplementary Fig. 2a). Again, our data re-address the importance of the free N-terminus after previous report of 2 or 5 extra amino acids, showing 20-fold or 150-fold slower catalytic efficiency^25^. In order to figure out how the glycine at the N-terminus affects protease activity, we crystallized the GM^pro^ protein and solved the crystal structure at a resolution of 2.0 Å. The overall structure of GM^pro^ is highly similar to those of SARS-CoV-2 native M^pro^ and SARS-CoV native M^pro^ (Supplementary Fig. 3a). The first residue Ser is disordered in GM^pro^ structure, indicating this residue cannot stabilize Glu166 of the S1 subsite in the neighboring protomer, which may be the main reason for the decrease of enzyme activity (Supplementary Fig. 3a). However, the extra glycine did not induce the main chain of residues 141–142 moves towards the S1 subsite as seen in previous report with five extra amino acids^25^ (Supplementary Fig. 3b).

### Enzyme inhibitory activity of Boceprevir and GC376

Boceprevir is a serine protease inhibitor that was approved by FDA to treat HCV infection in 2011. It was reported that the ketoamide group of Boceprevir can reversibly bind covalently to the Ser139 of HCV NS3/4A protease, whose hydroxyl group function as nucleophilic groups in enzymatic reaction^27^. Boceprevir was shown to be a time-dependent inhibitor with a fast-initial binding followed by a slow formation of the covalent adduct against NS3/4A protease^28^. GC376 is a cysteine protease covalent inhibitor against picornaviruses, noroviruses, and coronaviruses, and has shown promise in treating cats with fatal feline infectious peritonitis (FIP) caused by FIPV^29,30^. For time-dependent inhibitors, we tried to evaluate the equilibrium-binding constant *K*_i_ and the inactivation rate constant *k*_inac_ for covalent bond formation of Boceprevir and GC376 without preincubation. Progress curve of peptide hydrolysis by M^pro^ showed the inactivation processes of both two inhibitors are very slow (Supplementary Fig. 2b, c). So we used the IC_50_ values of these two inhibitors, 8.0 μM and 0.15 μM, to represent the inhibitory activity (Fig. 1d, f), which has also been used in other covalent inhibitor studys^31,32^. Meanwhile, we have performed the inhibition assay to test if these two compounds inhibit bovine chymotrypsin at the concentrations of 20 and 50 μM (Supplementary Fig. 2d). The results showed that GC376 does not have any inhibition activity against the bovine chymotrypsin, while Boceprevir shows very trivial inhibition activity, indicating the M^pro^ inhibitory activity of these two compounds are specific.

### Antiviral results of Boceprevir and GC376

**Fig. 2:**
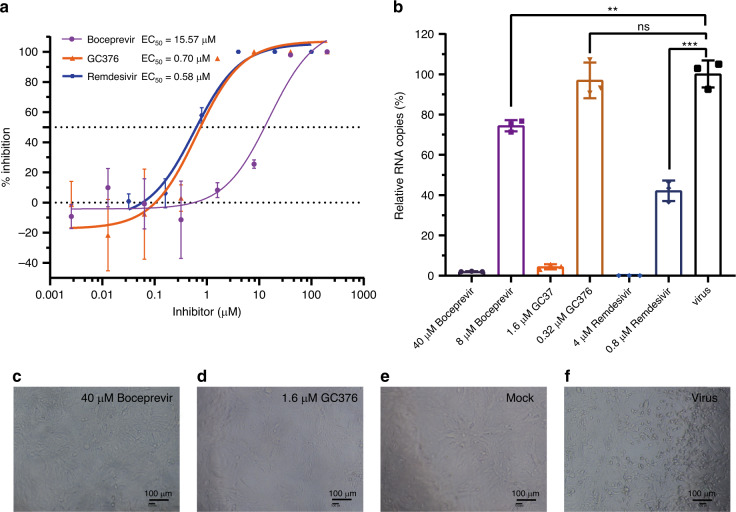
Inhibition of SARS-CoV-2 replication in Vero cells by Boceprevir and GC376. **a** The inhibitory effect of Boceprevir and GC376 treatment on SARS-CoV-2 replication. Remdesivir was used as a positive control. **b** The relative concentration of viral RNA present in the supernatant at 72h post infection determined by Real-time quantitative PCR analysis after Boceprevir and GC376 treatment, unpaired *t* test for comparison of relative viral RNA copies incubated with inhibitor vs virus control (*n* = 3 independent replicates). *p* = 0.0036, 0.6474, 0.0003 for 8 μM Boceprevir, 0.32 μM GC376, and 0.8 μM Remdesivir, respectively (ns: not significant *P* > 0.05, **P < 0.01, ***P < 0.001). **c**–**f** Virus-induced cytopathic effect in Vero cells after Boceprevir and GC376 treatment. Scale bar represents 100 μm. Error bars: mean ± S.D. of three independent replicates. Source data are provided as a source data file.

We further evaluated the inhibition effects of the two inhibitors on the replication of live virus. Both Boceprevir and GC376 showed effects against the SARS-CoV-2, with EC_50_ values of 15.57 μM and 0.70 μM, respectively (Fig. 2a). As a positive control, Remdesivir inhibited the SARS-CoV-2 replication with an EC_50_ value of 0.58 μM. No obvious pathological changes were observed when cells were incubated with 40 μM Boceprevir (Figs. 2c) or 1.6 μM GC376 (Fig. 2d) after infection. These compounds did not show obvious cytotoxicity at a concentration up to 200 μM in Vero cells (Supplementary Fig. 4). In order to further validate the antiviral effect of these inhibitors, we performed the traditional plaque assay stained with crystal violet. The plaque produced by the SARS-CoV-2 does not look like circles, which caused that it is difficult to quantify the antiviral efficacy for GC376 and Boceprevir. The possible reason is that the virus has been incubated with cells for a long time and multiple plaques are connected together. However, in presence of 40 μM Boceprevir or 1.6 μM GC376, the size and number of plaques are obviously smaller and less (Supplementary Fig. 5a, b). In addition, combination of 1 μM GC376 and 1 μM Remdesivir can completely inhibit viral replication in virus plaque assay (Supplementary Fig. 5d), showing additive effect of the joint application of RdRp inhibitors and protease inhibitors targeting different viral proteins.

### Inhibition mechanism of Boceprevir and GC376 against M^pro^

**Fig. 3:**
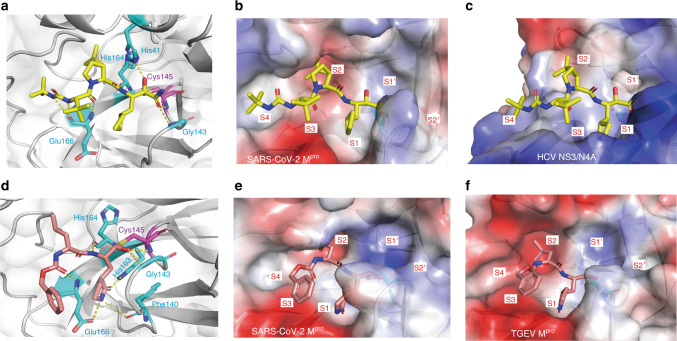
Structures of SARS-CoV-2 M^pro^ in complex with Boceprevir or GC376 and comparison with other structures. **a** Detailed interactions between Boceprevir (yellow) and SARS-CoV-2 M^pro^ (Cysteine protease, presented by cartoon and transparent surface). The amino acids involved were present by stick and colored by cyan. Hydrogen bond interactions were shown in dashed lines. Boceprevir created a covalent bond with Cys145 (purple). **b** The binding pocket of Boceprevir bound to SARS-CoV-2 M^pro^. M^pro^ is shown in surface representation. Boceprevir in the S1, S2, S3, and S4 positions of the active site of SARS-CoV-2 M^pro^ were labeled. **c** The binding pocket of Boceprevir bound to hepatitis C virus (HCV) NS3/4A serine protease (PDB: 2OC8). The S1, S2, S3, and S4 positions of the active site of HCV NS3/4A serine protease were labeled. Boceprevir created a covalent bond with Ser139. **d** Detailed interactions between GC376 (pink) and SARS-CoV-2 M^pro^. GC376 created a covalent bond with Cys145 (presented by stick and colored by purple). **e** The binding pocket of GC376 bound to SARS-CoV-2 M^pro^. GC376 in the S1, S2, and S3 positions of the active site of SARS-CoV-2 M^pro^ were labeled. **f** The binding pocket of GC376 bound to Transmissible gastroenteritis coronavirus (TGEV) M^pro^ (PDB: 4F49). GC376 in the S1, S2, and S3 positions of the active site of TGEV M^pro^ (present by charged surface) were labeled, and GC376 created a covalent bond with Cys144.

In order to elucidate the inhibitory mechanisms of these two compounds, we determined the crystal structures of M^pro^-Boceprevir and M^pro^-GC376 complexes at resolutions of 1.60 Å and 2.00 Å, respectively (Supplementary Fig. 6 and Supplementary Table 1). The unambiguous electron density maps show that the two inhibitors bind in the active site of M^pro^ in different conformations (Supplementary Fig. 7). In the M^pro^-Boceprevir complex structure, the Sγ atom of the nucleophilic Cys145 in M^pro^ forms a C–S covalent bond with the keto carbon of Boceprevir, which is a typical Michael addition (Fig. 3a, b and Supplementary Fig. 8a). Residues His41, Gly143, and His164 form four hydrogen-bonding interactions with the amide backbone of Boceprevir in one side, and residue Glu166 form three hydrogen-bonding interactions with Boceprevir in the other side. The small size of cyclobutylalanice (c-Bua) residue can be tolerated by S1 subsite. However, the P1 c-Bua residue has no interaction with S1 subsite (Fig. 3b). The hydrophilic Glu166 pushes the P1 hydrophobic group away and exposes the c-Bua residue to solvent. This can explain why Boceprevir displays a moderate activity against M^pro^. The rigid P2 dimethylcyclopropylproline (DMCP) residue can fit S2 subsite well (Fig. 3a) and has hydrophobic interaction with Met149 and Asp187 (Supplementary Fig. 8a). The hydrophobic P3 and P4 tert-butyl (tBu) residue can interact with Met165 in S3 subsite and Gln189, Gln192, Thr190 in S4 subsite (Supplementary Fig. 8a). Compared with HCV NS3/4A-Boceprevir complex^27^, the binding pocket are quite different due to low structural similarity, and the compound has large conformational changes to fit the HCV NS3/4A binding pocket (Fig. 3c). In all, 80% P1 c-Bua residue of Boceprevir is buried in S1 subsite which contributes the largest factor to binding. By contrast, in the SARS-CoV-2 M^pro^-Boceprevir complex structure, the P2 DMCP residue and P4 tBu cap are 80% buried other than P1 c-Bua residue. About 40% of P2, P3, and P4 residues are buried in S2, S3, and S4 subsite of HCV NS3/4A protease, which are highly exposed to solvent (Fig. 3c)^27^.

In the M^pro^-GC376 complex structure, the bisulfite group of GC376 was removed and GC376 created a covalent bond with Cys145 as aldehyde form in the complex structure (Fig. 3d, e and Supplementary Fig. 8b). The glutamine surrogate ring of GC376 fits into the S1 pocket and has hydrogen bonding interactions with the carboxyl group of Glu166. The Leu of GC376 fits into S2 hydrophobic pocket, which is consists of residues Arg40, His41, Met49, Tyr54, and Asp187. Compared with transmissible gastroenteritis virus (TGEV) M^pro^- GC376 complex, both the binding pocket and the configuration of GC376 are highly similar, suggesting a conserved interaction mode for GC376 against different coronaviruses (Fig. 3f). The crystal structures of GM^pro^-Boceprevir and GM^pro^-GC376 complexes at resolutions of 1.80 Å and 1.40 Å was also determined to evaluate the influences of the extra glycine at N terminal. Compared to M^pro^-Boceprevir, only P4 residue of Boceprevir has slight changes in GM^pro^-Boceprevir complex (Supplementary Fig. 3c). The binding mode of GC376 with GM^pro^ or M^pro^ has no obvious changes (Supplementary Fig. 3d).

## Discussion

We have performed the SAR analysis of other 3CLpro inhibitors reported recently. Compared with the inhibitors N3^26^, 13a (IC_50_ = 0.67 μM)^31^, 11a (IC_50_ = 0.053 μM), and 11b (IC_50_ = 0.04 μM)^32^, the P1 c-Bua residue is very important for inhibition activity. P2 moieties bulkier than cyclohexane of 13b and cyclopropane of 11a may be tolerant, which has been proved in the M^pro^-Boceprevir complex structure. Introduction of hydrogen bond acceptor F at P2 residue in 11b which has hydrogen bond interaction with Q189 can increase inhibition activity. The indole group of 11a/11b and the benzene ring group of GC376 showed that P3 is more suitable for aromatic conjugated group.

During our initial screen, there are four other HCV protease inhibitors which showed much lower activity against the main protease of SARS-CoV-2. Telaprevir, also a covalent inhibitor of serine protease^33^, loses inhibition activity against M^pro^ of SARS-CoV-2 which may be caused by steric hindrance of the large P2 quinoxaline moiety. The active pocket of HCV serine protease is larger and more solvent exposed than that of SARS-CoV-2 M^pro^ according to the complex structures of HCV serine protease co-crystallization with the inhibitors simeprevir, asunaprevir, and grazoprevir^34–36^. Therefore, the macrocyclic compound simeprevir, asunaprevir, and grazoprevir can not be accommodated in the relatively narrower pocket of SARS-CoV-2 M^pro^.

As we presented in the text, GC376 has more potent inhibition efficiency than Boceprevir, which makes GC376 might be more advantageous than Boceprevir in the clinical practice. However, GC376 has shown side effects such as retarded development of adult teeth in the animal tests^37^. The delayed eruption of some adult teeth was observed only in cats treated for 3 months or longer, not in cats treated for 2 weeks^38^. Therefore, GC376 has the potential to be a short-term treatment of COVID-19 for 1–2 weeks in combination with Remdesivir, because of COVID-19 is an acute disease.

In a word, the structural basis of interaction between the compounds and the main protease provides a good starting point for the optimization and design of more potent drugs against the COVID-19.

## Methods

### Protein expression and purification

To express the SARS-CoV-2 M^pro^ protein with an extra glycine residue at the N-terminus, the cDNA encoding residues 3264–3569 of *ORF1ab* (GenBank: MN908947.3) was synthesized and codon-optimized for expression in *E. coli*. The coding sequence was then cloned into the *Nco*I and *Not*I sites of the pET-52b vector (Genscript) with a N-terminal tag followed by TEV cleavage sequences. The recombinant protein was expressed in *E. coli* strain BL21 (DE3) as soluble proteins after inducing with 0.2 mM isopropyl-β-d-thiogalactopyranoside (IPTG) at an OD_600_ of 0.6-0.8 and expressing at 16 °C for 18 h. The cells were lysed by sonication in the lysis buffer (20 mM Tris, 50 mM NaCl, 20 mM imidazole, pH 8.0). After 19,802 × *g* centrifugation 30 min, the supernatants were then purified by affinity chromatography using the HisTrap HP 5 ml columns (GE Healthcare). The target protein was eluted with the elution buffer (20 mM Tris, 50 mM NaCl, 300 mM imidazole, pH 8.0). The purified proteins per mg were incubation with 2 μL reconstruction TEV protease (Solarbio, P2060) at 30 °C for 2 h after centrifugation. The hydrolyzed proteins were then purified by affinity chromatography using the HisTrap HP 5 ml columns (GE Healthcare) again and further purified by size-exclusion chromatography using a Hiload 16/600 Superdex 75 PG column (GE Healthcare) equilibrated with the binding buffer (20 mM Tris-HCl, 150 mM NaCl, 1 mM DTT, 1 mM EDTA, and pH 7.8). Expression and purification of the native SARS-CoV-2 M^pro^ protein were performed according to literature^26^. The recombinant protein was expressed in *E. coli* strain BL21 (DE3) as soluble proteins after inducing with 0.5 mM IPTG at an OD600 of 0.6–0.8 and expressing at 16 °C overnight. The cells were lysed by sonication in the lysis buffer (20 mM Tris, 50 mM NaCl, pH 8.0). After 19,802 × *g* centrifugation 30 min, the supernatants were then purified by affinity chromatography using the HisTrap HP 5 ml columns (GE Healthcare). The target protein was eluted with the elution buffer (20 mM Tris, 50 mM NaCl, 300 mM imidazole, and pH 8.0). The purified proteins per mg were incubation with 20 μL recombinant human rhinovirus protease (Genscript, Z03092) at 30 °C for 2 h after centrifugation. The hydrolyzed proteins were then purified by affinity chromatography using the HisTrap HP 5 ml columns (GE Healthcare) again and further purified by size-exclusion chromatography using a Hiload 16/600 Superdex 75 PG column (GE Healthcare) equilibrated with the binding buffer (20 mM Tris-HCl, 150 mM NaCl, 1 mM DTT, 1 mM EDTA, pH 7.8).

### Antiviral compounds

Saquinavir (Cat no. HY-17007), Ritonavir (Cat no. HY-90001), Indinavir (Cat no. HY-B0689), Nelfinavir Mesylate (Cat no. HY-15287A), Amprenavir (Cat no. HY-17430), Lopinavir (Cat no. HY-14588), Atazanavir sulfate (Cat no. HY-17367A), Fosamprenavir (Cat no. HY-78726), Tipranavir (Cat no. HY-15148), Darunavir (Cat no. HY-17040), Boceprevir (Cat no. HY-10237), Telaprevir (Cat no. HY-10235), Simeprevir (Cat no. HY-10241), Asunaprevir (Cat no. HY-14434), Grazoprevir (Cat no. HY-15298), Carfilzomib (Cat no. HY-10455), and Bortezomib (Cat no. HY-10227) were purchased from MedChemExpress. GC376 (Cat no. T5188) were purchased from TargetMol.

### Enzyme activity study

In all, 10 μL of 100 μM substrate solution (Dabcyl-TSAVLQ↓SGFRKMK-Edans) (Genscript) was added to black 96-well plate (Greiner) with 40 μL final concentration of 200 nM GM^pro^ or native M^pro^ in 25 mM Tris buffer (pH = 8.0). The relative fluorescence units (RFU) value was measured with an excitation wavelength of 360 nm and emission wavelength of 490 nm at 37 °C for 1 h by using SpectraMax Paradigm Muti-Mode Detection Platform (Molecular Devices, USA)^31^. Experiments were performed in triplicate. Then the progress curve of peptide hydrolysis was plotted by GraphPad Prism 8.0. First 1000 s change of fluorescence value was used to calculate the initial rate *v*_0_ by SoftMax Pro 7.1.

### M^pro^ enzyme activity inhibition test

Compounds in Table 1 were diluted in 25 mM Tris buffer (pH = 8.0) to a final concentration of 100 μM for screen. DMSO was used as a solvent control. In all, 10 μL compound solution was add to black 96-well plate (Greiner). In total, 30 μL of 2 μM GM^pro^ was added to the plate and incubated with the compounds at 37 °C incubator for 30 min. In total, 330 μL of 25 mM Tris buffer was also added as blank control. Then 10 μL of 20 μM peptide substrate (Dabcyl-TSAVLQ↓SGFRKMK-Edans) solution (Genscript) in DMSO was added. The RFU value was measured with an excitation wavelength of 340 nm and emission wavelength of 490 nm at 37 °C for 1 h by using a microplate reader (TECAN Infinite 200 Pro, Switzerland). The RFU change curves vs time with or without inhibitors were plotted by GraphPad Prism 8.0.

Because GC376 and Boceprevir are time-dependent covalent inhibitors, we evaluated the enzyme inhibitory activity without any preincubation. In all, 20 mM GC376 and Boceprevir in DMSO were diluted to 60 μM to 0.015 μM and 120 μM to 0.03 μM by 25 mM Tris buffer (pH = 8.0) respectively. In total, 30 μL inhibitor solution with a series of concentration in 25 mM Tris buffer (pH = 8.0) was mixed with 10 μL of 100 μM peptide substrate firstly. In total, 30 μL Tris buffer was also mixed with 10 μL of 100 μM peptide substrate as negative control. Then, 10 μL of 200 nM final concentration of M^pro^ was added to the plate. The RFU value was measured with an excitation wavelength of 360 nm and emission wavelength of 490 nm at 37 °C for 1 h by using SpectraMax Paradigm Muti-Mode Detection Platform (Molecular Devices, USA)^39^. Experiments were performed in triplicate. First 1200 s change of fluorescence value was used to calculate the reaction rate *v*_0_ by SoftMax Pro 7.1. The reaction rate of different inhibitor concentration is divided by the reaction rate of the negative control to calculate the inhibition rate with Microsoft Excel 2016. Inhibition curve was plotted by GraphPad Prism 8.0.

### Chymotrypsin enzyme activity inhibition test

In total, 10 μL final concentration of 50 μM, 20 μM of GC376, and Boceprevir in 25 mM Tris buffer (pH = 8.0) was mixed with 10 μL of 100 μM peptide substrate (Dabcyl-KATVRLQAGNATEE-Edans) solution (Genscript) in a black 96-well plate (Greiner). In all, 30 μL of 1 μM α-Chymotrypsin from bovine pancreas (Solarbio, C8660) in 25 mM Tris buffer (pH = 8.0) was then add to the plate. The RFU value was measured with an excitation wavelength of 360 nm and emission wavelength of 490 nm at 37 °C for 1 h by using SpectraMax Paradigm Muti-Mode Detection Platform (Molecular Devices, USA). Experiments were performed in triplicate. The progress curve of peptide hydrolysis was plotted by GraphPad Prism 8.0.

### Crystallization

In all, 8 mg/ml and 12 mg/ml GM^pro^ or M^pro^ (in 10 mM Tris, 1 mM EDTA, 1 mM DTT, pH 7.8) was incubated with 2 mM inhibitor at 4 °C for 18 h. All the crystals were obtained by using the sitting drop vapor diffusion method with 1 μL protein mixing with 1 μL reservoir solution and then equilibrating against 100 μL reservoir solution at 18 °C. The initial crystallization screenings were carried out using the commercially available kits. The native GM^pro^ was crystallized in 7% PEG6000, 100 mM MES (pH 6.1)^40^. The M^pro^-Boceprevir and GM^pro^-Boceprevir were crystallized in 20% PEG5000, 0.1 M BIS-TRIS (pH 6.5). While the M^pro^-GC376 and GM^pro^-GC376 complexes were crystallized in 14% PEG4000, 0.1 M MES monohydrate (pH 6.0) and 10% PEG6000, 0.1 M MES (pH 6.1), and 3% DMSO respectively.

### Data collection and structure determination

Diffraction data were collected at Shanghai Synchrotron Radiation Facility (SSRF) BL17U (wavelength, 0.97918 Å). For data collection, the crystals were cryo-protected by briefly soaking in reservoir solution supplemented with 20% (v/v) glycerol before flash-cooling in liquid nitrogen. The dataset was processed with HKL2000 software^41^. The native M^pro^ structure was determined by the molecular replacement method using Phaser^42^ with the previously reported SARS-CoV M^pro^ structure (PDB code, 3F9F), while the complexes were further determined with the solved M^pro^ structure. The atomic models were completed with Coot^43^ and refined with phenix.refine in Phenix^44^, and the stereochemical qualities of the final models were assessed with MolProbity^45^. Data collection, processing, and refinement statistics are summarized in Supplementary Table 1. All structural figures were generated using Pymol software (http://www.pymol.org).

### Cells and viruses

African green monkey kidney Vero cells were maintained in DMEM (Gibco, C11995500BT) supplemented with 10% fetal bovine sera (FBS) (Gibco, A31608-02), 200 mg/ml streptomycin, and 200 IU/ml penicillin (Gibco, 15140122) at 37 °C. SARS-CoV-2 was isolated from Wuhan seafood market by China CDC. All the infection experiments were performed in a biosafety level-3 (BLS-3) laboratory.

### Cell viability assay

The Vero cells were seeded in 96-well plate and cultured overnight. In total, 100 μL different concentration of compound solution in DMEM was added to the Vero cells after PBS (Gibco, C10010500BT) wash and incubated for 48 h at 37 °C, 5% CO_2_. Meanwhile, 100 μL DMEM was added to the Vero cells as negative control. In total, 10 μL CCK8 assays (TargetMol, C0005) was added to each well directly. OD_450_ was measured by using a microplate reader (TECAN Infinite 200 Pro, Switzerland) after 3 h incubation at 37 °C. Experiments were performed in triplicate. OD_450_ value in present of different concentration of compound is divided by the OD_450_ value of the negative control to calculate the percentage cytotoxicity with Microsoft Excel 2016. The cytotoxicity curve was plotted by GraphPad Prism 8.0.

### In vitro antiviral assays

In all, 20 mM GC376 and Boceprevir in DMSO were diluted to 200 μM to 0.00256 μM by DMEM contained 1% FBS. In total, 10 mM remdesivir in DMSO were diluted to 100 μM to 0.032 μM. DMSO medium (1%) was used as negative control. Vero cells cultured overnight in 96-well plate, were infected by 0.01 MOI virus for 2 h. The medium was removed, and fresh inhibitor-containing medium was added to the cells then. Experiments were performed in quadruplicate. After 48 h, the cell in triplicate was lysed in lysis buffer. Viral RNA was extracted from 100 μL supernatant of infected cells using the automated nucleic acid extraction system (TIANLONG, China), following the manufacturer’s recommendations. SARS-CoV-2 detection was performed using the One Step PrimeScript RT-PCR kit (TaKaRa, Japan) on the LightCycler® 480 Real-Time PCR system (Roche, Rotkreuz, Switzerland). *ORF 1ab* was amplified from cDNA and cloned into MS2-nCoV-*ORF1ab* and used as the plasmid standard after its identity was confirmed by sequencing. A standard curve was generated by determination of copy numbers from serially dilutions (103-109 copies) of plasmid. The following primers used for quantitative PCR were *ORF1ab*-F: 5′-AGAAGATTGGTTAGATGATGATAGT-3′, *ORF1ab*-R: 5′-TTCCATCTCTAATTGAGGTTGAACC-3′, and probe 5′-FAM-TCCTCACTGCCGTCTTGTTGACCA-BHQ1-3′. In all, 72 h later, the cytopathic effect changes of the rest replicate were observed by microscope.

The Ct value was changed to virus copy number (10^*y*^) by formula *y* = (Ct-35.461)/−3.4546 with Microsoft Excel 2016. The virus copy number in percent of different concentration inhibitors is divided by that of negative control to get percentage inhibition or percentage relative RNA copies with Microsoft Excel 2016. The inhibition curve was plotted by fitting log(inhibitor) vs. response (three parameters) mode with GraphPad Prism 8.0. The EC_50_ was also calculated in the table of results by GraphPad Prism 8.0. The column graph of relative RNA copies in percent of inhibitors and unpaired *t* test between two columns were performed by GraphPad Prism 8.0.

### Plaque-reduction assays

In all, 2 × 10^5^ Vero cells were seeded in a 24-well plate and cultured overnight in 24-well plate. The cells were infected by 200 μL SARS-CoV-2 (100 PFU) for 2 h. The medium was then removed, and fresh medium (DMEM contained 2% FBS and 1.2% Avicell) containing appropriate concentrations of inhibitors was added to the cells. The cells were incubated for 72 h at 37 °C with 5% CO_2_. The overlay was discarded. The cells were fixed for 30 min with 4% polyoxymethylene (Solarbio, P1110) and stained with crystal violet working solution for 15 min.

### Statistical analysis and reproducibility

The *p* values described in this study were calculated by unpaired *t* test using GraphPad Prism 8.0. Figures 2c–f and Supplementary Fig. 5a–e have been repeated twice and similar results were observed.

### Reporting summary

Further information on research design is available in the Nature Research Reporting Summary linked to this article.

## Supplementary information

Supplementary File

Peer Review File

Reporting Summary

## Data Availability

Figures 1a, b, d, f and 2a, b; Supplementary Figs. 2 and 4 have associated raw data in this paper. Supplementary files are available from the corresponding authors upon request. Atomic coordinates and structure factors have been deposited in the Protein Data Bank under accession codes PDB 7BRO, PDB 7BRP, PDB 7BRR, PDB 7C6U, and PDB 7C6S. Source data are provided with this paper.

## Source data

Source Data
