# Supplementary material for: Both Boceprevir and GC376 efficaciously inhibit SARS-CoV-2 by targeting its main protease
Source: Nat Commun. 2020 Sep 4;11:4417. doi: 10.1038/s41467-020-18233-x (PMC7474075; doi:10.1038/s41467-020-18233-x)
Supplement: Supplementary file 1 — Supplementary File [file 41467_2020_18233_MOESM1_ESM.pdf]

Supplementary Information for:

# Both Boceprevir and GC376 efficaciously inhibit SARS-CoV-2 by targeting its main protease

Fu et al.

Supplementary Table 1. Diffraction data and refinement statistics

	GM^{pro}	GM^{pro} -Boceprevir	GM^{pro} -GC376	M^{pro} -Boceprevir	M^{pro} -GC376
**PDB Code**	7BRO	7BRP	7BRR	7C6S	7C6U
**Data collection**					
Space group	C2	P21	P21	C2	C2
Cell dimensions					
a, *b*, *c* (Å)	113.43, 54.26, 44.96	47.37, 53.55, 113.77	55.45, 99.02, 59.58	97.23, 80.25, 54.48	100.00, 79.61, 51.85
α, β, γ (°)	90, 100.67, 90	90, 101.66, 90	90,108.54, 90	90,116.72, 90	90,114.71,90
Wavelength (Å)	0.979	0.979	0.979	0.979	0.979
Resolution (Å)	50-2.20 (2.07-2.00)	50-1.80 (1.86-1.80)	50-1.40 (1.50-1.40)	50-1.60 (1.66-1.60)	50-2.00 (2.07-2.00)
R_{merge}	0.021 (0.057)	0.053 (0.611)	0.042 (0.1.085)	0.084 (0.792)	0.055 (0.227)
$I / \sigma I$	70.7 (30.9)	28.4 (2.8)	43.2 (1.9)	22.3 (2.5)	34.4 (9.2)
CC1/2	0.998 (0.994)	0.998 (0.887)	0.998 (0.712)	0.991 (0.897)	0.993 (0.985)
Completeness (%)	99.6 (100.0)	99.5 (99.3)	100.0(100.0)	99.9 (100.0)	99.8 (100.0)
Redundancy	5.8 (5.9)	6.0 (5.9)	6.8 (6.6)	7.5 (7.6)	7.5 (7.5)
**Refinement**					
Resolution (Å)	25.83-2.00	27.57-1.80	29.03-1.40	48.67-1.60	43.26-2.00
No. reflections	18253	49507	108002	45351	24895
R_{work} / R_{free}	0.2533/0.2991	0.2156/0.2396	0.1887/0.1973	0.2075/0.2222	0.24.15/0.2512
No. atoms					
Protein	2341	4658	4733	2341	2329
Ligand/ion	0	74	58	37	29
Water	122	384	575	347	180
B-factors					
Protein	30.9	25.7	22.8	20.0	34.4
Ligand/ion		19.0	33.8	19.1	28.6
Water	34.3	29.1	32.4	26.3	30.9
R.m.s. deviations					
Bond lengths (Å)	0.002	0.003	0.006	0.007	0.002
Bond angles (°)	0.604	1.253	0.836	0.758	0.502
Ramachandran plot					
Favored (%)	96.64	97.99	98.16	97.66	97.32
Allowed (%)	3.36	2.01	1.84	2.34	2.68
Outliers (%)	0	0	0	0	0

*Values in parentheses are for highest-resolution shell.

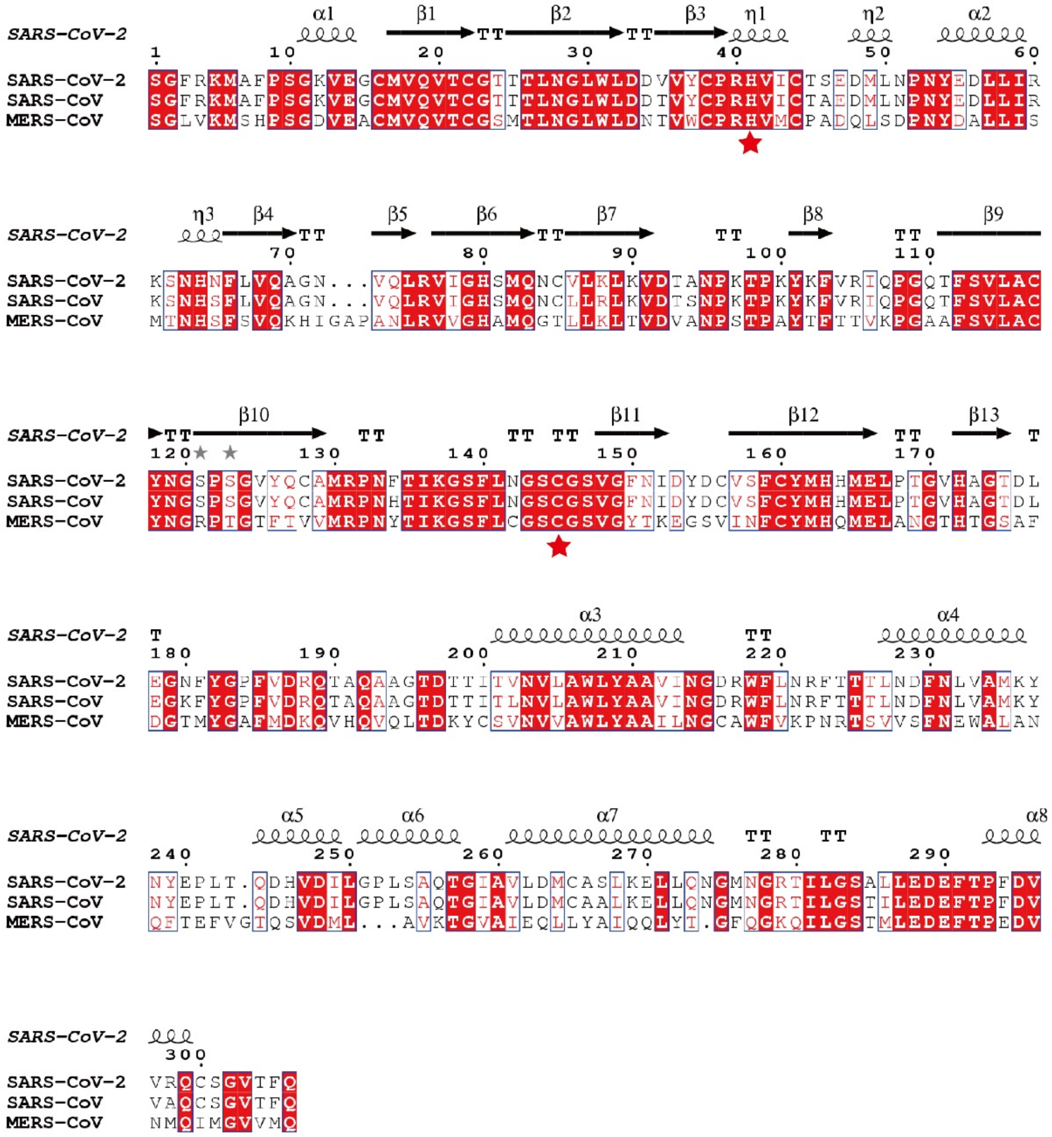

Supplementary Figure 1. Sequence alignment of M^{pro} from SARS-CoV-2, SARS-CoV and MERS-CoV. The conserved catalytic residues (His41 and Cys145, in SARS-CoV-2) are indicated using red stars.

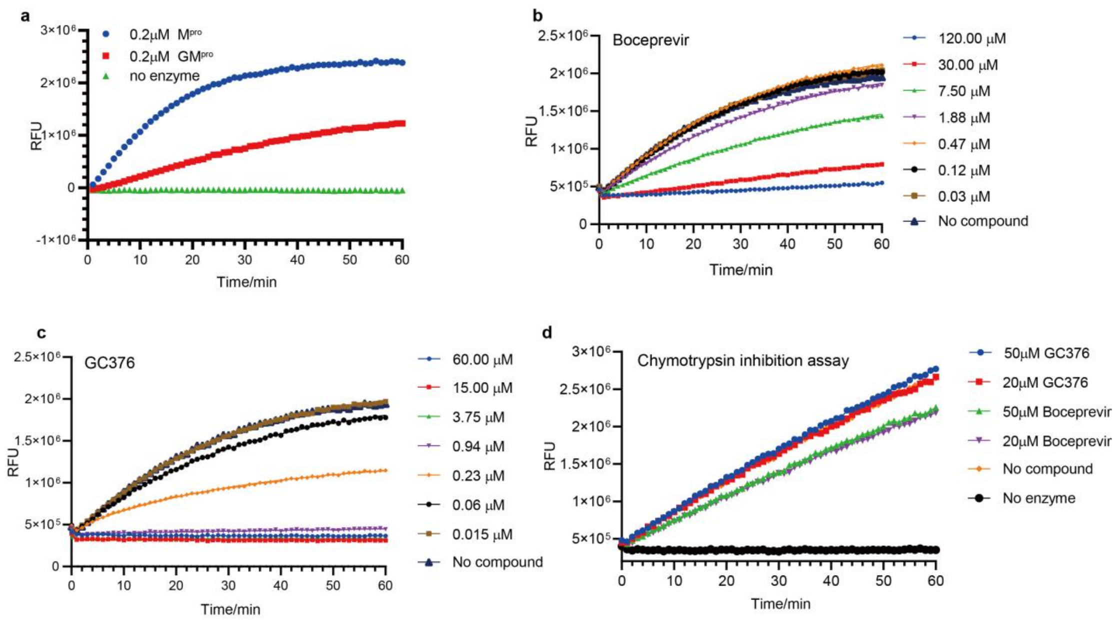

Supplementary Figure 2. the progress curve of peptide hydrolysis by SARS-CoV-2 M^{pro} and chymotrypsin.

a, Comparation of enzyme activity between GM^{pro} and M^{pro}. **b, c,** M^{pro} inhibition curve by Boceprevir and GC376. Slow inactivation of M^{pro} activity by Boceprevir and GC376 was observed from the curve. **d,** Bovine α-chymotrypsin inhibition curve by GC376 and Boceprevir. Source data are provided as a source data file.

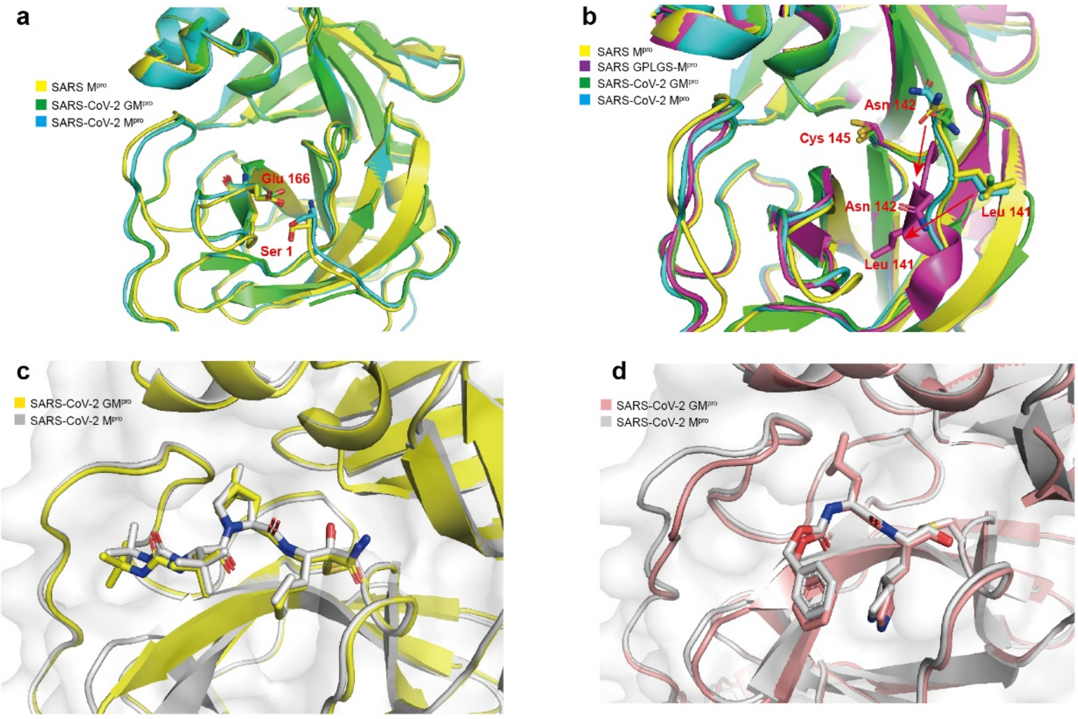

Supplementary Figure 3. Structural details of the enzyme active site in M^{pro}.

a, b, N-terminus structure and enzyme active site of GM^{pro} (colored by green) merged with native SARS-CoV-2 M^{pro} (colored by cyans, 6Y2E), native SASR virus M^{pro} (colored by yellow, 2H2Z) and GPLGS taged SASR virus M^{pro} (colored by purple, 1UJ1) crystal structure reported previously. **c,** GM^{pro} cocrystal structure (colored by yellow) with Boceprevir merged with M^{pro} cocrystal (colored by gray). **d,** GM^{pro} cocrystal structure with GC376 (colored by pink) merged with M^{pro} cocrystal (colored by gray).

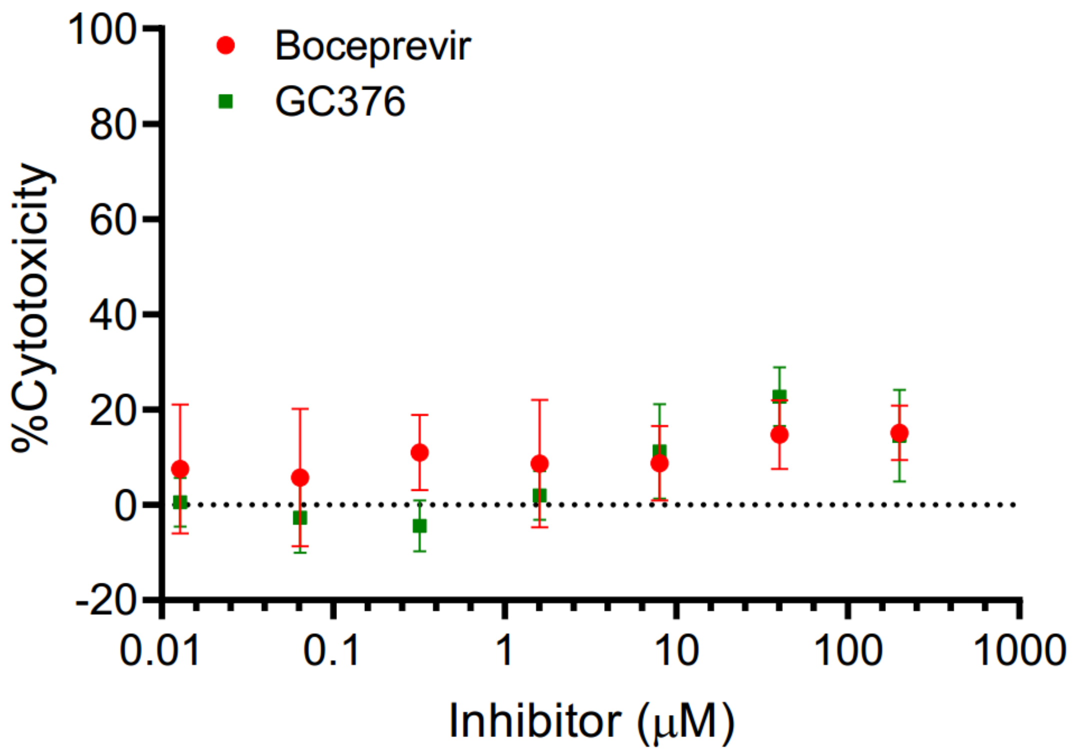

Supplementary Figure 4. Both Boceprevir and GC376 show no cellular toxicity to Vero cells at test concentrations. Cytotoxicity of Boceprevir and GC376 in Vero cells were evaluated by CCK-8 assay. Error bars: mean ± S.D. of three independent replicates. Source data are provided as a source data file.

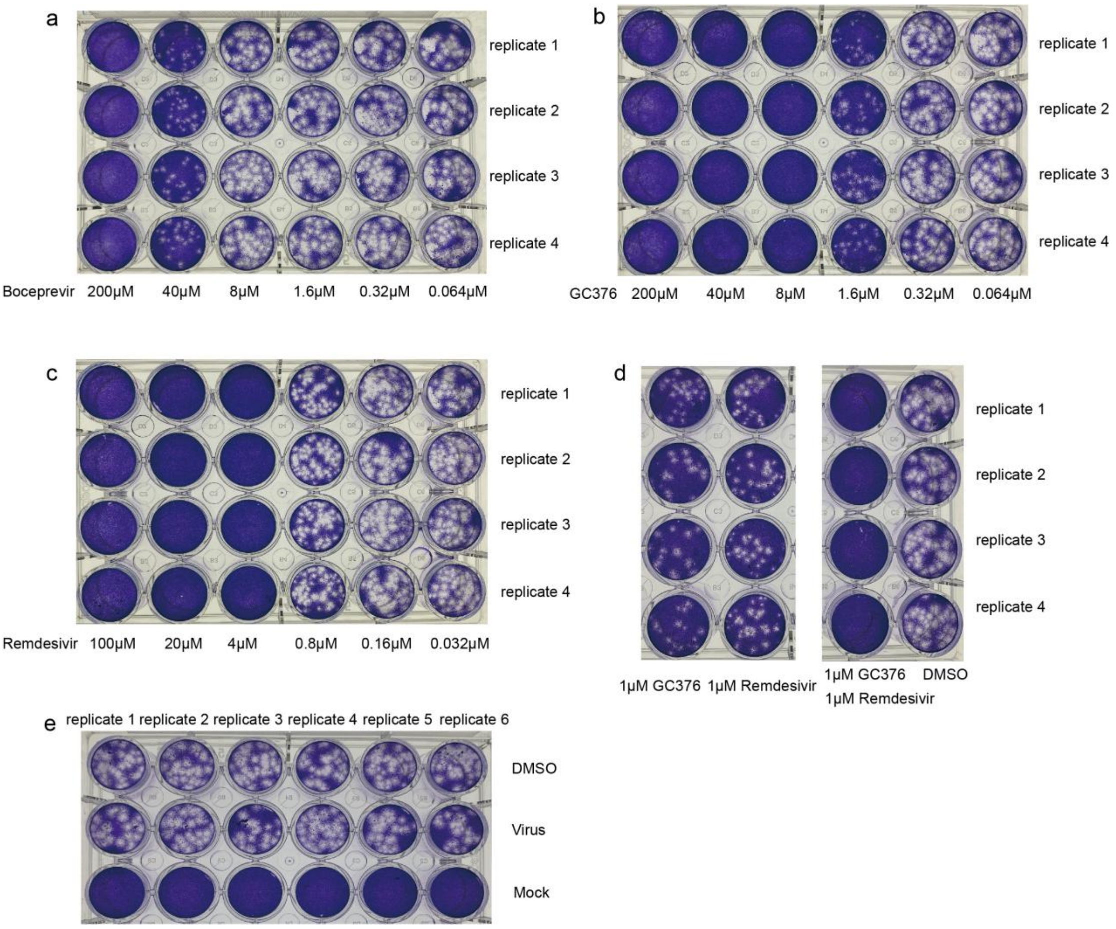

Supplementary Figure 5. Images for the plaque-reduction assay

a, The inhibitory effect of Boceprevir in different concentration. **b**, The inhibitory effect of GC376 in different concentration. **c,** The inhibitory effect of Remdesivir in different concentration. **d**, Combine inhibitory effect of Remdesivir and GC376. **e,** DMSO means that 1% DMSO medium was incubated with Vero cells after virus infection. Virus means that medium without inhibitors or DMSO was incubated with Vero cells after virus infection. Mock means that the Vero cells was not infected with virus.

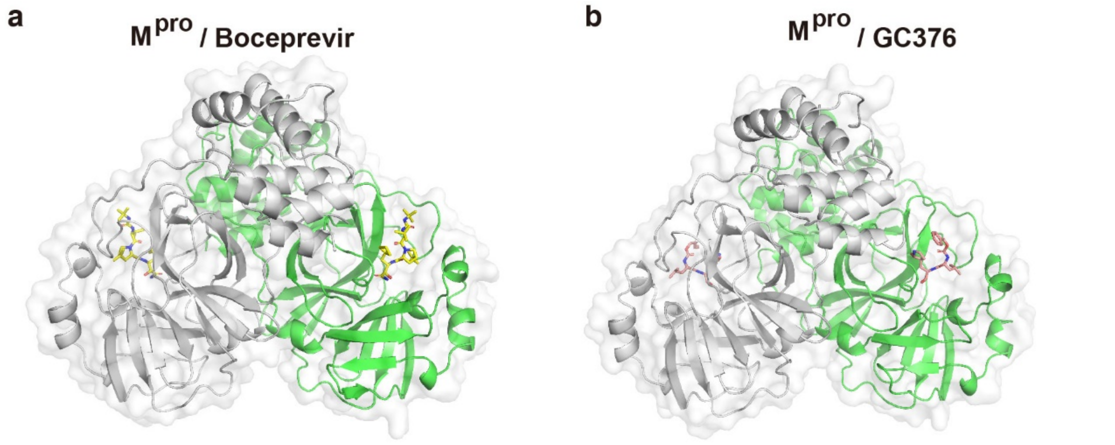

Supplementary Figure 6. Overall structures of SARS-CoV-2 M^{pro} complexed with Boceprevir or GC376.

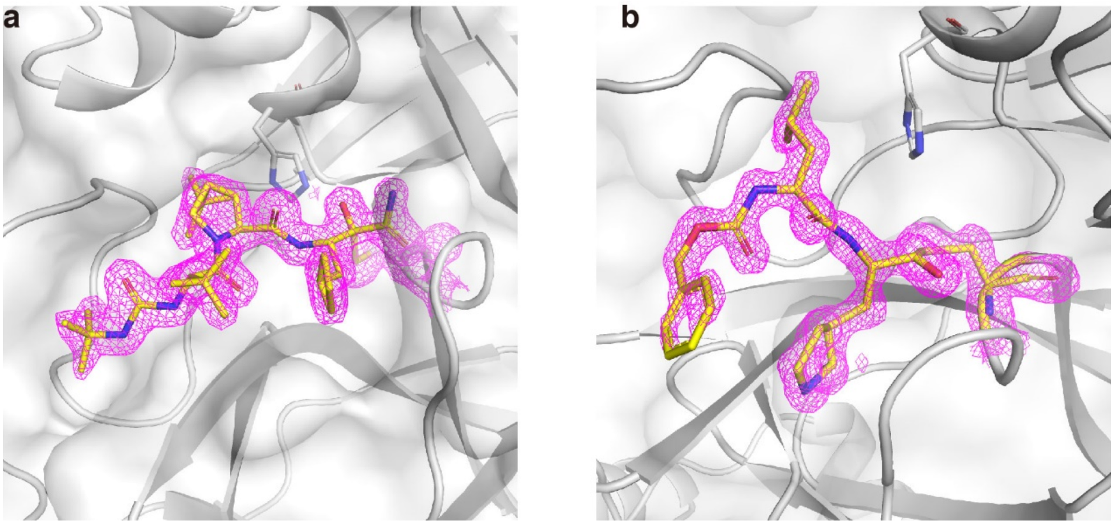

Supplementary Figure 7. Electron density of SARS-CoV-2 M^{pro} complexed with Boceprevir (a) and GC376 (b). The panels show portions of 2Fo-Fc electron density maps for the two compounds contoured at 1.5 sigma.

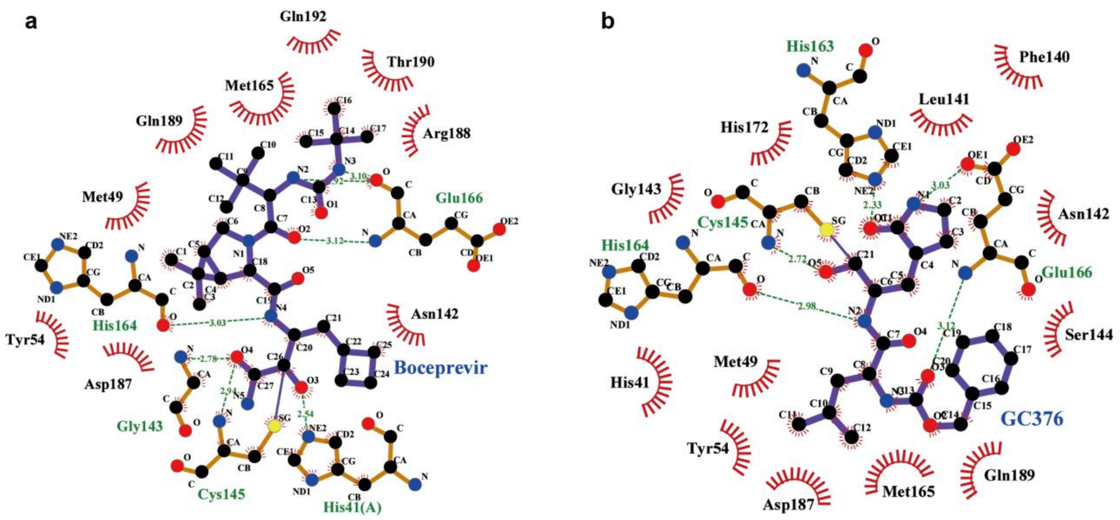

Supplementary Figure 8. The detailed interactions of Boceprevir and GC376 bound to SARS-CoV-2 M^{pro}. Protein side chains and the inhibitors are shown as ball and sticks. Hydrogen bonds are shown as green dotted lines. The covalent bond between inhibitors and Cys145 residue is in purple. Spoked arcs represent nonbonded contacts. Figure is generated using LigPlot+ diagram[1].

Supplementary References

1. Laskowski, R. A. & Swindells, M. B. LigPlot+: multiple ligand-protein interaction diagrams for drug discovery. *J Chem Inf Model* **51**, 2778-2786 (2011).
