# Supplementary material for: Both Boceprevir and GC376 efficaciously inhibit SARS-CoV-2 by targeting its main protease
Source: Nat Commun. 2020 Sep 4;11:4417. doi: 10.1038/s41467-020-18233-x (PMC7474075; doi:10.1038/s41467-020-18233-x)
Supplement: Supplementary file 3 — Reporting Summary [file 41467_2020_18233_MOESM3_ESM.pdf]

nature research

Corresponding author(s): George Fu Gao

Last updated by author(s): Jul 8, 2020

Reporting Summary

Nature Research wishes to improve the reproducibility of the work that we publish. This form provides structure for consistency and transparency in reporting. For further information on Nature Research policies, see our Editorial Policies and the Editorial Policy Checklist.

Statistics

For all statistical analyses, confirm that the following items are present in the figure legend, table legend, main text, or Methods section.

n/a	Confirmed	
☐	☒	The exact sample size (*n*) for each experimental group/condition, given as a discrete number and unit of measurement
☐	☒	A statement on whether measurements were taken from distinct samples or whether the same sample was measured repeatedly
☐	☒	The statistical test(s) used AND whether they are one- or two-sided<br>*Only common tests should be described solely by name; describe more complex techniques in the Methods section.*
☒	☐	A description of all covariates tested
☒	☐	A description of any assumptions or corrections, such as tests of normality and adjustment for multiple comparisons
☐	☒	A full description of the statistical parameters including central tendency (e.g. means) or other basic estimates (e.g. regression coefficient) AND variation (e.g. standard deviation) or associated estimates of uncertainty (e.g. confidence intervals)
☐	☒	For null hypothesis testing, the test statistic (e.g. *F*, *t*, *r*) with confidence intervals, effect sizes, degrees of freedom and *P* value noted<br>*Give P values as exact values whenever suitable.*
☒	☐	For Bayesian analysis, information on the choice of priors and Markov chain Monte Carlo settings
☒	☐	For hierarchical and complex designs, identification of the appropriate level for tests and full reporting of outcomes
☒	☐	Estimates of effect sizes (e.g. Cohen's *d*, Pearson's *r*), indicating how they were calculated

Our web collection on statistics for biologists contains articles on many of the points above.

Software and code

Policy information about availability of computer code

Data collection	Data collection in related enzyme activity study SoftMax Pro 7.1, Data collection in crystal study: HKL2000, Data collection in : Microsoft Excel 2016, Data collection in Real-Time PCR: LightCycler® 480 Software.
Data analysis	Data analysis in crystal study: Phaser, MolProbity, PHENIX 1.16, CCP4 7.1, WinCoot 0.8.2, Pymol 2.4, LigPlot+. Data analysis in related enzyme activity study, cell viability assay and antiviral assays: GraphPad Prism 8.0, Microsoft Excel 2016.

For manuscripts utilizing custom algorithms or software that are central to the research but not yet described in published literature, software must be made available to editors and reviewers. We strongly encourage code deposition in a community repository (e.g. GitHub). See the Nature Research guidelines for submitting code & software for further information.

Data

Policy information about availability of data

All manuscripts must include a data availability statement. This statement should provide the following information, where applicable:

- Accession codes, unique identifiers, or web links for publicly available datasets
- A list of figures that have associated raw data
- A description of any restrictions on data availability

- Atomic coordinates and structure factors have been deposited in the Protein Data Bank under accession codes PDB 7BRO [https://doi.org/10.2210/pdb7BRO/pdb], PDB 7BRP [https://doi.org/10.2210/pdb7BRP/pdb], PDB 7BRR [https://doi.org/10.2210/pdb7BRR/pdb], PDB 7C6U [https://doi.org/10.2210/pdb7C6U/pdb] and PDB 7C6S [https://doi.org/10.2210/pdb7C6S/pdb].
- Fig. 1 a, b, d, f; Fig. 2 a and b; Supplementary Fig. 2 and 4 have associated raw data in this paper.
- The data supporting the findings of this study are available in the manuscript and supplementary files are available from the corresponding authors upon request.

Field-specific reporting

Please select the one below that is the best fit for your research. If you are not sure, read the appropriate sections before making your selection.

- [x] Life sciences
- [ ] Behavioural & social sciences
- [ ] Ecological, evolutionary & environmental sciences

For a reference copy of the document with all sections, see nature.com/documents/nr-reporting-summary-flat.pdf

Life sciences study design

All studies must disclose on these points even when the disclosure is negative.

Sample size	No sample size calculation was performed. 3 independent biological replicates were used in this study because it is common in the biological experiment.
Data exclusions	There are no data excluded from the analyses.
Replication	Experiments were performed in triplicate, expect plaque-reduction assays were performed in quadruplicate. all attempts at replication were successful.
Randomization	Randomization is not relevant to this study.
Blinding	The compounds was marked as number when we performed screen assay and antiviral assay. For other experiments, the investigators were not blinded to group allocation during data collection or analysis. Because blinding has no effect on the experimental results.

Reporting for specific materials, systems and methods

We require information from authors about some types of materials, experimental systems and methods used in many studies. Here, indicate whether each material, system or method listed is relevant to your study. If you are not sure if a list item applies to your research, read the appropriate section before selecting a response.

Materials & experimental systems

n/a	Involved in the study	
x		Antibodies
	x	Eukaryotic cell lines
x		Palaeontology and archaeology
x		Animals and other organisms
x		Human research participants
x		Clinical data
x		Dual use research of concern

Methods

n/a	Involved in the study	
x		ChIP-seq
x		Flow cytometry
x		MRI-based neuroimaging

Eukaryotic cell lines

Policy information about cell lines

Cell line source(s)	All cell lines were obtained from the American Type Culture Collection (ATCC).
Authentication	no cell authentication method was used.
Mycoplasma contamination	no contamination observed.
Commonly misidentified lines (See ICLAC register)	Vero E6 is a commonly misidentified lines.
