# Supplementary material for: Both Boceprevir and GC376 efficaciously inhibit SARS-CoV-2 by targeting its main protease
Source: Nat Commun. 2020 Sep 4;11:4417. doi: 10.1038/s41467-020-18233-x (PMC7474075; doi:10.1038/s41467-020-18233-x)
Supplement: Supplementary file 2 — Peer Review File [file 41467_2020_18233_MOESM2_ESM.pdf]

Editorial Note: This manuscript has been previously reviewed at another journal that is not operating a transparent peer review scheme. The manuscript was considered suitable for publication without further review at Nature Communications.
